# A rare case of extraintestinal amebiasis

**DOI:** 10.1186/s12879-022-07348-9

**Published:** 2022-04-11

**Authors:** Bao Fu, Jinjing Wang, Xiaoyun Fu

**Affiliations:** 1 Department of Critical care Medicine, Affiliated Hospital of Zunyi Medical University, Dalian Road 149, Zunyi City, 563003 Guizhou China; 2 Department of Pathology, Affiliated Hospital of Zunyi Medical University, Zunyi City, 563003 Guizhou China

**Keywords:** Amebiasis, Urinary amoebiasis, Central nervous system, Pleuropulmonary

## Abstract

**Background:**

Amoebiasis is caused by the protozoan *Entamoeba histolytica*, which is a rare infectious disease in developed countries. If the trophozoites enter the blood, it can spread through the body, such as brain, and lungs. Cases of simultaneous infection of multiple organs are extremely rare.

**Case presentation:**

Here we report a case of simultaneous infection of amoeba in pulmonary pleura, urinary system and central nervous system. Although the patient received anti amoeba treatment, the prognosis of the patient was poor.

**Conclusions:**

In this patient, multiple extraintestinal amebic infections in the absence of clinically confirmed intestinal amebiasis or amebic liver abscess are rare and pose diagnostic challenges. The disseminated amebiasis has significantly increased the mortality. Early diagnosis and appropriate treatment may reduce the mortality of disseminated amebiasis.

## Background

Amoebiasis is caused by the protozoan *Entamoeba histolytica* (*E. histolytica*), which is distributed throughout the world [1, 2]. Amoebiasis kills 40,000–100,000 people annually and is the fourth leading cause of death from protozoan infection [3]. Amoebiasis may present as asymptomatic or mild to severe symptoms, including abdominal pain, diarrhea, or bloody diarrhea [4]. Some patients may develop invasive extraintestinal disease. The most common extraintestinal manifestation is an amoebic liver abscess [5]. In some rare cases, amebiasis may affect the lungs, heart, brain, kidneys, spleen, and skin [5, 6]. Today, with effective treatment, the mortality rate for patients with uncomplicated disease is less than 1%. However, in 5–10% of cases, amebic liver abscesses may be complicated by abdominal rupture, potentially increasing mortality. The mortality rate of amebic pericarditis and pulmonary amebiasis exceeds 20% [5]. As a result, disseminated amebiasis has significantly increased mortality. Here, we reported a very rare case of a patient with extraintestinal amoebic lesions involving multiple organs.

## Case presentation

**Fig. 1:**
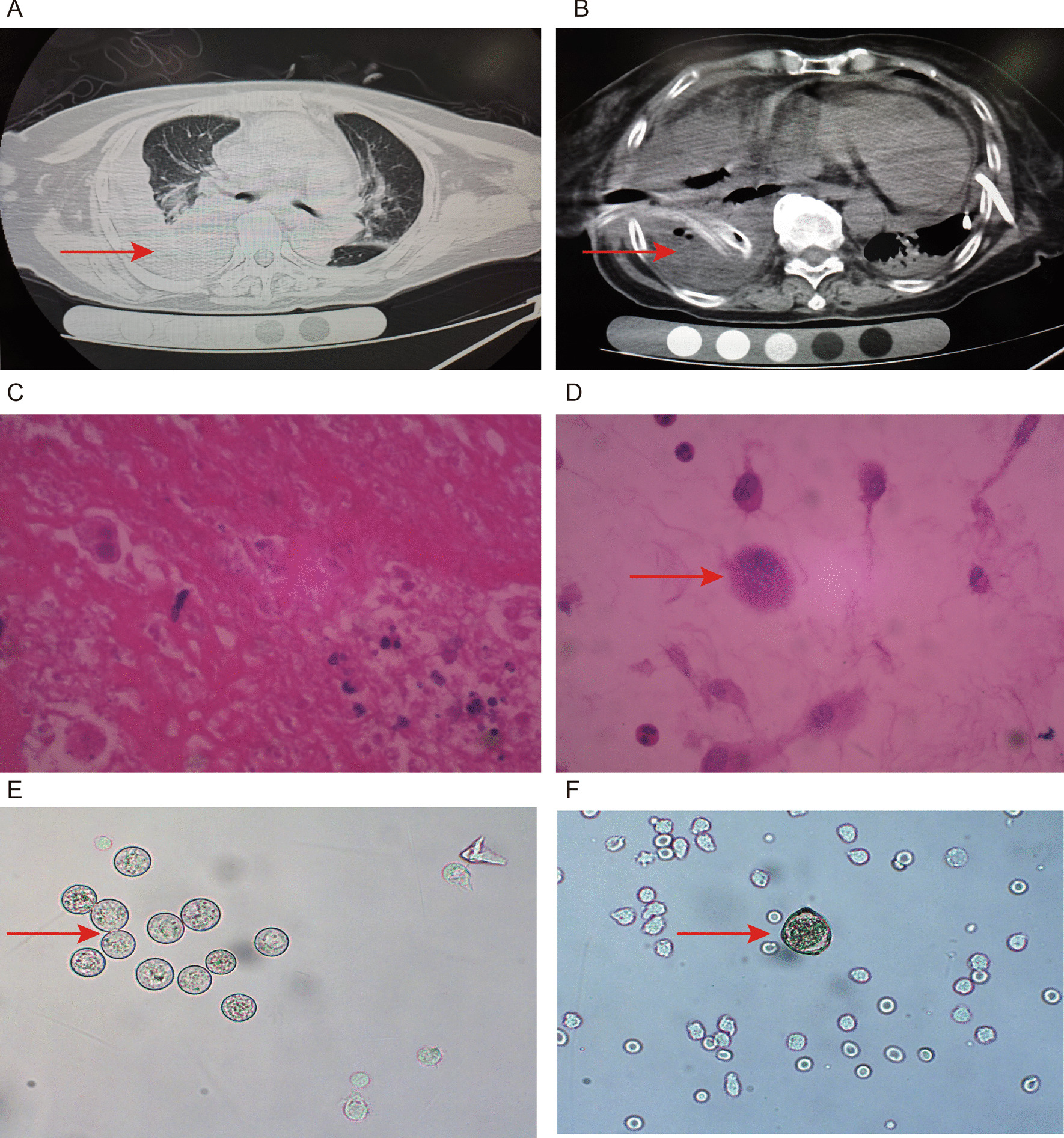
Chest CT on the second day after admission (**A** and **B**). Pleural tissue biopsy and pleural fluid microscopy (**C** and **D**). Microscopic examination of urine and cerebrospinal fluid (**E** and **F**)

A 75 year old female patient was admitted to Affiliated Hospital of Zunyi Medical University for “fatigue, chest tightness and shortness of breath for 3 months”. This hospital is a tertiary teaching hospital located in Guizhou Province. The patient developed fever (38.0–38.5 °C) 3 months ago, accompanied by fatigue, chest tightness, without cough and abdominal pain. The patient believed that otitis media was onset and did not seek medical treatment. She was a history of cholecystitis and bilateral renal cysts. Physical examination showed temperature: 36.6 °C heart rate: 106/min, respiratory rate: 16/min, blood pressure: 122/80 mmHg, SPO_2_ 92% (room air). Chest CT at admission showed double lung pneumonia, bilateral pleural effusion and pericardial effusion (Fig. 1A and B). Pleural effusion is yellow turbid liquid. Laboratory test results of pleural effusion: rivalta test (+ +), total cell count: 5030 × 10^6^/L, nucleated cell count: 4000 × 10^6^/L (normal range: < 300 × 10^6^/L), total protein: 59.9 g/L (normal range: < 25 g/L), glucose: 4.44 mmol/L (normal range: 3.6–5.5 mmol/L), lactate dehydrogenase:350 U/L (normal range: 0–200 U/L). White blood cell (WBC): 2.78 × 10^9^/L (normal reference range: 3.5–9.5 × 10^9^/L); neutrophil: 2.09 × 10^9^/L (1.8–6.3 × 10^9^/L); lymphocyte: 0.47 × 10^9^/L (1.1–3.2 × 10^9^/L); eosinophils: 0.03 × 10^9^/L (0.02–0.52 × 10^9^/L); C-reactive protein (CRP): 2.30 mg/L (0.068–8.2 mg/L). CD4 cell absolute count: 68.0/μL (normal reference range: 414–1440 μL), CD8 cell absolute count: 104.0/μL (normal reference range: 238–1250/μL), CD3 cell absolute count: 184.0/μL (normal reference range: 770–2860/μL). This patient lives in a third-tier city in China, located in the Yunnan-Kweichow Plateau, a typical karst landform. There are sporadic cases of amoebic infection in the area.

The patient’s residence is a high incidence area of tuberculosis. She was suspected of tuberculous pleurisy and received anti-tuberculosis treatment (Moxifloxacin sodium chloride injection, 0.4 g, ivgtt, qd; isoniazid0.3 g, p.o. qd; rifampicin, 0.45gmg, p.o. qd; pyrazinamide, 1.25 g, p.o. qd; ethambutol hydrochloride, 0.75 g, p.o. qd). After 2 weeks of treatment in hospital, the patient's symptoms of fatigue and shortness of breath improved temporarily, and the patient was discharged. The patient continued to take anti-tuberculosis drugs after discharge. However, no acid-fast bacilli were found in the sputum and pleural effusion of the patient during hospitalization.

Five months after discharge, the patient was hospitalized again due to “fever, chest tightness and fatigue”. At this admission, the patient's chest CT showed bilateral pleural effusion. Patient blood T-SPOT of tuberculosis was negative. After detailed asking of history, the patient complained that he had a eating raw river shrimp experience 10 years ago. Because the anti-tuberculosis treatment was ineffective, we examined the patient’s pleural effusion under microscope. The suspiciousamebic trophozoites were found in her pleural effusion. The haematoxylin and eosin (H&E) staining demonstrated scattered amebic trophozoites. (Fig. 1C and D). The Subsequent PCR testing of pleural effusion deposits also confirmed *E. histolytica*. She immediately received metronidazole 500 mg intravenously (q8h). After metronidazole treatment for 1 week, the patient developed diarrhea symptoms, which was yellow stool. No amoebic trophozoites were found on stool microscopy. After metronidazole treatment for 2 week, she developed psychiatric symptoms and drowsiness during metronidazole treatment. No brain abscess was found in multiple head CT examinations. She then underwent a lumbar puncture and direct wet mount observed 2 motile trophozoites (Fig. 1F). Amoeba protozoa were also found in the patient's urine (Fig. 1E). The subsequent PCR testing of cerebrospinal fluid and urine were also showed *E. histolytica*. Despite the implementation of anti-amoeba treatment for 3 weeks, the patient's condition still worsened. Because the patient suffered some side effects such as nausea, vomiting and unconsciousness during metronidazole infusion, his family asked to stop metronidazole. Unfortunately, the patient died 1 month after admission.

## Discussion and conclusions

Amoebic pleural effusions and abscesses are generally considered to be easily treatable with drainage and antimicrobial therapy [7]. This patient was initially misdiagnosed as tuberculous pleurisy and was ineffective after 2 weeks of anti-tuberculosis treatment. Symptoms of amebic pleural effusion improved with drainage, which may have contributed to the misdiagnosis of this patient. Another factor leading to the misdiagnosis of this patient is that she lives in an area with high incidence of tuberculosis. The diagnostic value of Escherichia histolytica in fecal specimens is limited. Detection of amebic antibodies may help be useful for early diagnosis. Detection of Entamoeba DNA in pus or sputum may be a sensitive and specific method.

*Entamoeba histolytica* is a human protozoan that induces amebiasis. Amoeba exists in two forms: infectious non-dividing cysts and actively dividing invasive trophozoites. Infected cysts are usually found in contaminated food and water. This patient may have been misdiagnosed because there was no evidence of intestinal amebiasis or amebic liver abscess. It is reported that only 10% of individuals exposed to the pathogen develop “symptomatic” aggressive amoebiasis; most cases are asymptomatic in the early stages or manifest as self-limiting mild diarrhea [8]. Extraintestinal manifestations of amoebic dysentery can occur along three routes: extension from the involved gastrointestinal tract, hematogenous or lymphogenous spread from the primary site, or direct contact with contaminated food or water [9]. It is reported that amoeba can infect the thoracic cavity [10], urethra [11], liver [12] and brain [13]. In this case, it is possible that the trophozoites enter the blood by invading the mesentery and then spread to other organs.

Extraintestinal amebiasis occurs more frequently and is the main reason of parasite death in developing countries [14]. The most common manifestation of extraintestinal amebiasis is amebic liver abscess. The most common symptoms of amebic liver abscess are fever, abdominal pain and hepatomegaly [15]. Pleuropulmonary complications often occur when a liver abscess is present. In all cases of liver abscess, pleural pulmonary complications occur in 20–35% of cases [16]. However, no liver abscess was found in this patient. Pleural chest pain and cough are frequent clinical manifestations of pleuropulmonary amoebiasis. A common imaging abnormality is a small to moderate right pleural effusion due to liver infiltration under the right diaphragm. Amoebiasis of the genitourinary system is a rare parasitic disease [17]. The rupture of an infected liver abscess around the kidney, through the blood circulation or from the lymphatic system of the kidney and urinary system are the two main routes of transmission of genitourinary amoebiasis [18, 19]. Free-living amoeba causes two rare central nervous system infections: primary amoebic meningoencephalitis and granulomatous amoebic encephalitis [20]. CT manifestations of amoebic infection of the central nervous system: panamoebic meningoencephalitis and granulomatous amoebic encephalitis [20]. There was no obvious lesion on CT of this patient, but amoeba protozoa was found in the cerebrospinal fluid. The clinical manifestations of extraintestinal amoebiasis are diverse and easy to be misdiagnosed. If the patient has a history of intestinal amebiasis and the extraintestinal organs are involved, it should be highly suspected of extraintestinal amebiasis. Asymptomatic *E. histolytica* infection may pose challenges for the diagnosis of extraintestinal amebiasis. Diagnosis of amebiasis usually relies on parasitological, molecular biology and immunological techniques. Microscopic examination of parasites in stool, body fluids, or tissue samples is considered an easy method of diagnosis [21].

Although many cases of amoebiasis have been reported in the past, cases of simultaneous infection of multiple organs are very rare. The main treatment for symptomatic amebiasis requires hydration and the use of metronidazole and/or tinidazole. The dose of metronidazole for adults is 500 mg orally every 6–8 h for 7–14 days. The adult dose of tinidazole is 2 g orally per day for 3 consecutive days. The prognosis of cases of amoebic meningoencephalitis is very poor, and deaths occur in most cases [13]. Various types of drugs and their combinations have been tested, but the prognosis is still poor. In recent years, the combined application of amphotericin B, fluconazole, rifampicin, azithromycin, dexamethasone and miltrexine in the treatment of primary amoebic meningoencephalitis [13]. Invasive amebic infections are treated with an amebic tissue active agent (tinidazole or metronidazole) followed by a lumen encapsulicide (dioxane or paromomycin) to eradicate colonization. A 5–10 days of treatment is used for amebic colitis and 10 days for amebic liver abscess [22]. Currently, there is no consensus on the treatment course of brain abscess. It is described in the literature for up to 8 weeks [23, 24].

This patient developed disseminated infection, which included pleuropulmonary, urinary system and central nervous system. Host factors, such as youth, immune status, genetic susceptibility, pregnancy, corticosteroid therapy, malnutrition and alcohol abuse, are considered to be risk factors for the transmission and severity of amebiasis [25, 26]. Our patient was malnourished and immunocompromised at admission, which may contribute to the greater invasiveness of amebiasis. The genotype and phenotype of the parasite, along with individual’s immune status and environmental factors, together determine the prognosis of infection with *E. histolytica* [25].

This patient lives in a high incidence area of tuberculosis and lacks evidence of intestinal amoeba and Amoeba liver abscess. Hence, it is easy to be misdiagnosed as tuberculous pleurisy. The misdiagnosis of tuberculous pleurisy, which resulted in the patient not receiving anti-amebic therapy as early as possible, may be one of the reasons for the deterioration of the patient’s condition.

## Data Availability

The authors stated that all the data and materials were true and available in the study.

## Abbreviation

CT: Computed tomography
